# Rift Valley Fever Outbreak during COVID-19 Surge, Uganda, 2021

**DOI:** 10.3201/eid2811.220364

**Published:** 2022-11

**Authors:** Caitlin M. Cossaboom, Luke Nyakarahuka, Sophia Mulei, Jackson Kyondo, Alex Tumusiime, Jimmy Baluku, Gloria Grace Akurut, Dianah Namanya, Kilama Kamugisha, Hildah Tendo Nansikombi, Alex Nyabakira, Semei Mutesasira, Shannon Whitmer, Carson Telford, Julius Lutwama, Stephen Balinandi, Joel Montgomery, John D. Klena, Trevor Shoemaker

**Affiliations:** Centers for Disease Control and Prevention, Atlanta, Georgia, USA (C.M. Cossaboom, S. Whitmer, J. Montgomery, J.D. Klena, T. Shoemaker);; Makerere University Department of Biosecurity, Ecosystems and Veterinary Public Health, Kampala, Uganda (L. Nyakarahuka);; Uganda Virus Research Institute, Entebbe, Uganda (L. Nyakarahuka, S. Mulei, J. Kyondo, A. Tumusiime, J. Baluku, J. Lutwama, S. Balinandi);; Uganda Wildlife Authority, Kampala (G.G. Akurut, D. Namanya, K. Kamugisha);; Uganda Public Health Fellowship Program, Kampala (H.T. Nansikombi, A. Nyabakira);; Case Hospital, Kampala (S. Mutesasira)

**Keywords:** Rift Valley fever, viral hemorrhagic fever, zoonotic, Bunyavirales, outbreak investigation, zoonoses, vector-borne infections, Uganda

## Abstract

Rift Valley fever, endemic or emerging throughout most of Africa, causes considerable risk to human and animal health. We report 7 confirmed Rift Valley fever cases, 1 fatal, in Kiruhura District, Uganda, during 2021. Our findings highlight the importance of continued viral hemorrhagic fever surveillance, despite challenges associated with the COVID-19 pandemic.

Rift Valley fever (RVF), a zoonotic mosquitoborne disease of livestock caused by Rift Valley fever virus (RVFV), is endemic throughout most of Africa and the Arabian Peninsula (*1*,*2*). Humans can be infected with RVFV through contact with blood, body fluids, products from infected livestock, or bites from infected mosquitoes (*1*,*3*). No human-to-human transmission has been documented (*4*). In humans, infections are typically asymptomatic or result in mild influenza-like illness (*1*). Severe illness, including hemorrhagic manifestations, occurs in ≈1%–2% of cases; the case-fatality rate among severe cases is ≈10%–20% (*1*,*5*). No approved human vaccine or specific treatment is available, but early supportive care may prevent complications and decrease death (*1*). In livestock, RVFV infection can cause abortions and high mortality, leading to substantial economic losses (*1*,*6*). We describe a fatal human case of RVF and the subsequent investigation and identification of 6 additional cases in Kiruhura District, Uganda, in 2021. We also note the role a COVID-19 surge played in delayed testing and patient care.

## The Study

**Figure 1:**
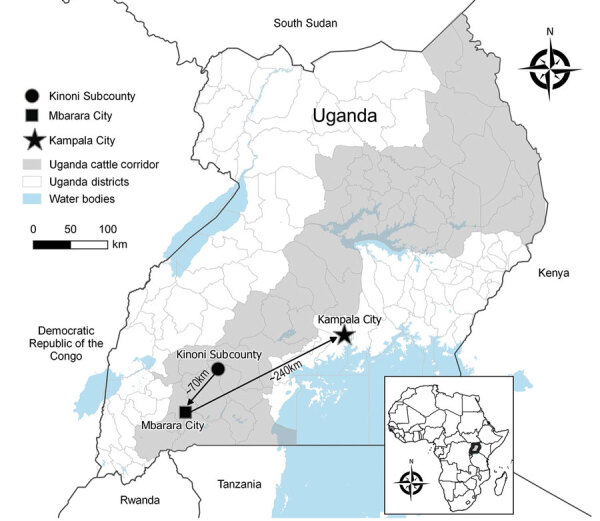
Locations where the index case-patient of a Rift Valley fever outbreak in Kinoni Subcounty sought care during the period of acute illness preceding her death, Uganda, 2021. Arrows indicate route patient followed during attempts to find diagnosis and care. Inset shows location of Uganda in Africa.

On May 7, 2021, fever, headache, fatigue, arthromyalgia, nausea, vomiting, and diarrhea developed in a previously healthy woman 19 years of age (P1), who sought treatment at a private clinic in Kinoni Subcounty, Kiruhura District, Uganda (Figure 1). She was treated empirically for malaria with no improvement. On May 9, after hematemesis developed, she sought treatment at and was admitted to another local private clinic. On May 11, the patient was transferred to the regional referral hospital in Mbarara District for further disease management (Figure 1). Anuric acute kidney injury, chest pain, and respiratory distress complicated her hospital course. She was transferred by ambulance the same day to the national referral hospital in Kampala (Figure 1) for critical care but was not admitted because the hospital had no available dialysis unit. She was subsequently referred to a nearby nonprofit private hospital but was not admitted because the intensive care unit was at capacity with patients with COVID-19. During transfer, P1’s clinical status deteriorated, and her hemorrhagic signs worsened.

On May 12, she was admitted to a private tertiary hospital with fever (38.0°C), jaundice, epistaxis, ecchymoses, gingival hemorrhage, respiratory distress, hypotension, focal seizures, and altered mentation. At admission, she was thrombocytopenic and anemic with deranged liver and renal function and electrolyte abnormalities (Table). The clinical team suspected a viral hemorrhagic fever (VHF) and collected blood for testing at the Uganda Virus Research Institute (UVRI). The patient died on May 13. 

**Table:** Selected results of hemogram and blood chemistry tests for specimen collected from case-patient 1 a day before she died of Rift Valley fever, Uganda, 2021

Selected tests	Absolute value	Reference range
Platelets, 10^3^/μL	60.00	138–475
Hemoglobin, g/dL	6.7	>12
Albumin, g/L	27.5	37–52
Total protein, g/L	50.7	68–90
Total bilirubin, μmol/L	109.32	5.13–32.49
Direct bilirubin, μmol/L	68.71	0.00–6.84
Alkaline phosphatase, U/L	313	47–160
Gamma-glutamyl transferase, U/L	173	8.0–41.3
Aspartate transferase, U/L	>913	11.4–28.8
Prothrombin time, s	15.8	10–13
Internal normalized ratio	1.1	<1.1
Creatinine, μmol/L	1098.33	44.2–79.6
Urea, μmol/L	>44.60	2.7–7.1
Sodium, mmol/L	112	135–146
Potassium, mmol/L	8.4	3.5–5.5
Calcium, mmol/L	1.24	2.20–2.65

UVRI testing confirmed RVFV infection by real-time reverse transcription PCR (rRT-PCR) (*7*) and IgM and IgG ELISA (*3*,*5*). On May 14, UVRI reported the confirmed case to the Uganda Ministry of Health.

Until she became ill, P1 resided with 8 family members in a rural area of Kinoni Subcounty. The week after RVFV was confirmed, a team from the Uganda Public Health Fellowship Program conducted interviews with the deceased woman’s family. The family owns large cattle herds that graze in pasture areas surrounding their homestead and reported that in the weeks before the woman’s illness, their cattle had appeared unwell; 1 had died and several had experienced abortions. Several goats from a neighboring farm had also reportedly aborted recently. The family reported that P1 had regularly milked the family’s cattle and that the family, including P1, had regularly consumed raw milk from the herd.

A male family member 20 years of age (P2), who lived ≈1 km away from P1, experienced signs and symptoms beginning May 28. On June 1, he sought treatment at a local health center with fever, headache, cough, nausea, abdominal pain, and hematemesis. A malaria rapid diagnostic test was negative. P2 was treated with paracetamol, promethazine, and ciprofloxacin and was discharged. RVF was immediately suspected because of increased awareness following P1’s diagnosis, so the clinical team collected a blood sample the same day and sent it to UVRI for VHF testing using the National Laboratory sample transport system (*8*). On June 2, melena and gingival hemorrhage developed, and P2 sought care at a private clinic, where he was admitted for supportive care. His symptoms improved overnight, and he was discharged the next day.

On June 3, a field team from UVRI and the Centers for Disease Control and Prevention (CDC) interviewed and collected blood samples for RVFV testing from 20 persons living in or around P1’s homestead that were willing to participate. The average age of participants was 28.6 years (range 9–67 years); 65% were male. Two participants, including P2, reported symptoms consistent with RVF at time of sampling and were tested using RVFV rRT-PCR and serology. The initial sample collected from P2 by the clinical team on June 1 was delayed in transit and not delivered to UVRI until June 8, but it eventually tested positive by rRT-PCR and IgM and IgG ELISA, as did the second sample collected from P2 by the field team on June 3. The other symptomatic participant tested negative. The remaining asymptomatic participants were tested by RVFV serology only; 2 were IgM and IgG positive, and 3 were IgM negative and IgG positive.

**Figure 2:**
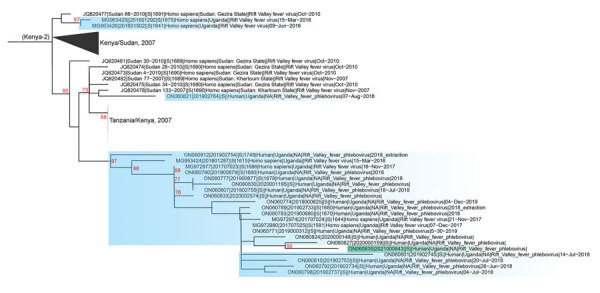
Phylogenetic analysis of Kenya-2 clade Rift Valley fever virus small segment from an outbreak in Uganda, 2021, compared with available full-length segments from GenBank (accession numbers shown). Green shading indicates sequence from Uganda outbreak; blue shading indicates historic RVFV sequences from Uganda. Red numbers indicate nodes with bootstrap support >70%. Complete phylogenies of small and large segments are shown in the Appendix.

We conducted next-generation sequencing (NGS) and phylogenetic analysis on the rRT-PCR positive sample from P1 (Figure 2; Appendix). Sequencing generated complete large and small segments but only a partial medium segment. The large and small segments (deposited into GenBank under accession nos. ON060834–5) were members of the Kenya-2 clade and clustered with a Uganda-specific sublineage collected during 2016–2020. The most closely related sequences were collected from the Wakiso and Kyankwanzi districts, both 181 km from Kiruhura District, during February–March 2020. This finding suggests the strain represented by the sequence isolated from P1 had wide geographic and temporal circulation in Uganda and might be endemic. We did not attempt NGS on samples from P2 because the rRT-PCR cycle thresholds suggested it would be unsuccessful; the first sample from P2 was likely degraded from lack of cold chain continuity during delayed transportation, and the second sample was collected late in the course of illness.

## Conclusion

We report 7 RVFV infections, 4 recent infections (positive by IgM testing, rRT-PCR, or both) and 3 past infections (IgG positive only), identified May–June 2021 in Kiruhura District, Uganda. The western region of Uganda, including Kiruhura District, is within the cattle corridor (Figure 1) and at high risk for RVF and Crimean-Congo hemorrhagic fever because of large livestock populations (*9*,*10*). The RVF case-patient who died was a young, previously healthy resident of a farming community with a history of contact with cattle and drinking raw milk from a herd with reported manifestations compatible with RVF.

In April–June 2021, Uganda experienced a second surge of COVID-19, leading to a nationwide lockdown in June 2021 (*11*), which likely delayed RVF recognition and care provision to P1, contributing to her death. P1 traveled >300 km in 5 days seeking care at 6 healthcare facilities (Figure 1) before a VHF was suspected only hours before she died. In addition, the specimen transport system, slowed by COVID-19 demands, delayed RVF confirmation for P2. 

Implications of delayed recognition and diagnosis could have been far worse with other VHFs endemic to western Uganda with higher case-fatality rates (e.g., Ebola, Marburg, and Crimean-Congo hemorrhagic fever viruses) (*9*,*12*,*13*), which unlike RVFV are capable of human-to-human transmission. Our findings highlight the critical need to improve access to diagnostics, renewed community and clinician education about VHFs in humans and animals, and improved surveillance and awareness of the continued threat of VHFs in Uganda and the region. 

Appendix Additional information from report of Rift Valley fever outbreak in Uganda.
