# Supplementary material for: Rift Valley Fever Outbreak during COVID-19 Surge, Uganda, 2021
Source: Emerg Infect Dis. 2022 Nov;28(11):2290–3. doi: 10.3201/eid2811.220364 (PMC9622231; doi:10.3201/eid2811.220364)
Supplement: Appendix — Additional information from report of Rift Valley fever outbreak in Uganda. [file 22-0364-Techapp-s1.pdf]

Article DOI: https://doi.org/10.3201/eid2811.220364

Rift Valley Fever Outbreak during COVID-19 Surge, Uganda, 2021

Appendix

Material and Methods

RNA Extraction, NGS Library Preparation, Bioinformatics and Phylogenetics

RNA was extracted from blood or serum specimens with MagMax in the High Containment Laboratory at Uganda Virus Research Institute, Uganda. RNA was DNase-treated and NGS libraries were made using NEBNext Ultra II Directional RNA library prep kit. Library was run on a iSeq 300 cycle cartridge. Consensus genomes were constructed using 1) de novo assembly and blasting of contigs to identify the closest reference sequence on GenBank, followed by iterative mapping of reads and contigs to closest reference sequence; and 2) iterative mapping of reads and contigs to ON060778–80, 201900879 and ON060813–5, 201902760 genomes, which are representative members of 2 separate RVFV clades in Uganda. Consensus genomes were called from bam files with the best read coverage using iVar (version 1.3, -m 2 -n N) (*1*). To build phylogenetic trees, all available full length RVFV segments were downloaded from GenBank, aligned using MAFFT (v7.450) and trees were constructed with RAxML (version 7.3.0, -m GTRGAMMA -p $RANDOM -f a -x $RANDOM -N 1000). Trees were visualized with ggtree and clades labeled according to Grobbelaar et al. (2011) and Samy et al. (2017) (*2*,*3*). Genbank accession no. ON060834–5.

References

1. Grubaugh ND, Gangavarapu K, Quick J, Matteson NL, De Jesus JG, Main BJ, et al. An amplicon-based sequencing framework for accurately measuring intrahost virus diversity using PrimalSeq and iVar. Genome Biol. 2019;20:8. PubMed https://doi.org/10.1186/s13059-018-1618-7

2. Grobbelaar AA, Weyer J, Leman PA, Kemp A, Paweska JT, Swanepoel R. Molecular epidemiology of Rift Valley fever virus. Emerg Infect Dis. 2011;17:2270–6. PubMed https://doi.org/10.3201/eid1712.111035

3. Samy AM, Peterson AT, Hall M. Phylogeography of Rift Valley fever virus in Africa and the Arabian peninsula. PLoS Negl Trop Dis. 2017;11:e0005226. PubMed https://doi.org/10.1371/journal.pntd.0005226

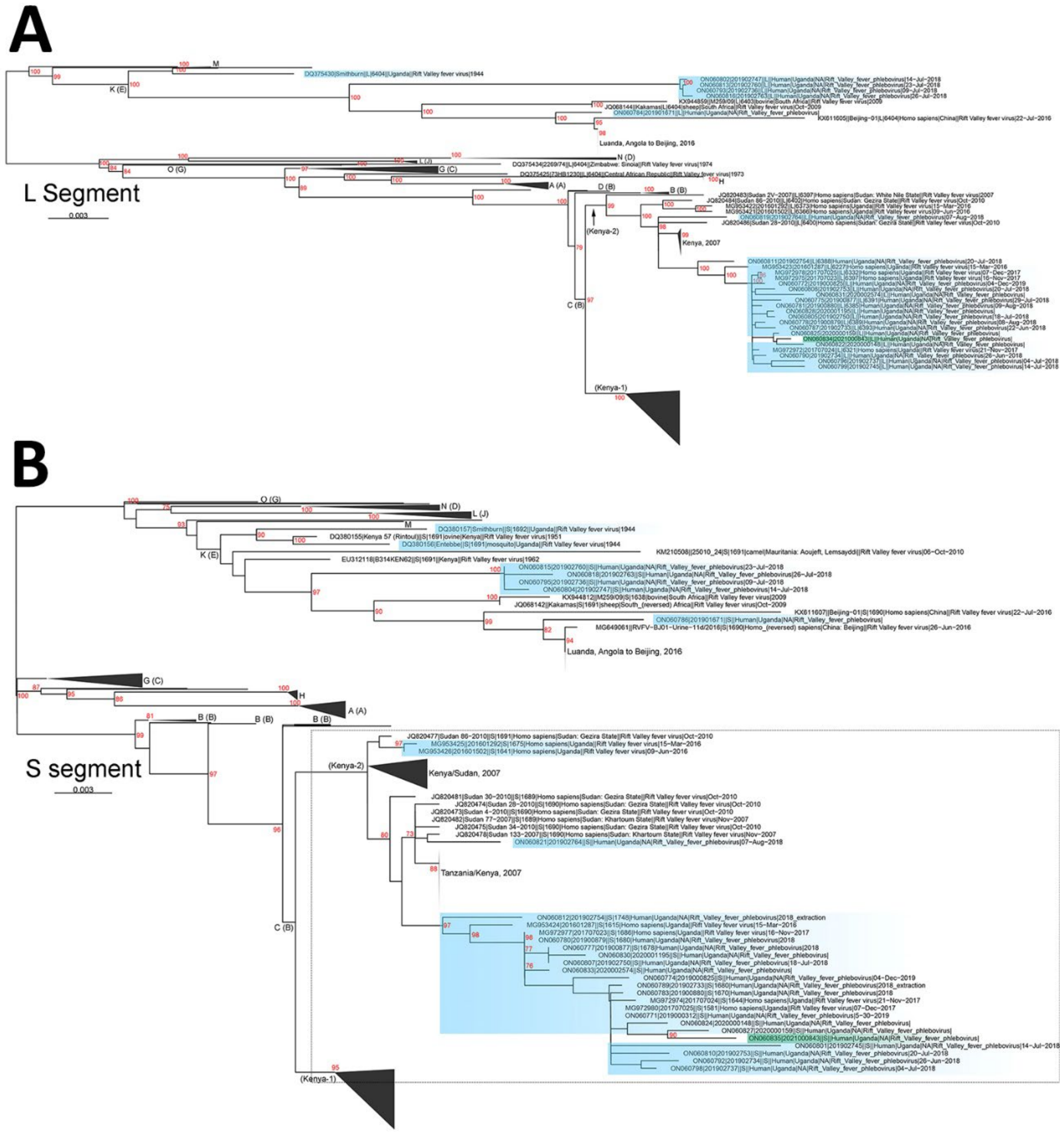

Appendix Figure. Phylogenetic analysis of all available full-length Rift Valley fever virus L and S segments from Genbank. Major clades are labeled according to previous publications (2,3). Nodes with bootstrap support >70% are labeled in red. A) Phylogenetic tree of RVFV L segment. B) Phylogenetic tree of RVFV S segment. Historic RVFV sequences from Uganda are shown in blue highlight and new sequence in green highlight in both panels.
